# MRI-Based Quantification of Pan-Alimentary Function and Motility in Subjects with Diabetes and Gastrointestinal Symptoms

**DOI:** 10.3390/jcm12185968

**Published:** 2023-09-14

**Authors:** Davide Bertoli, Esben Bolvig Mark, Donghua Liao, Tina Okdahl, Serena Nauser, Louise Hostrup Daugberg, Christina Brock, Birgitte Brock, Filip Krag Knop, Klaus Krogh, Jens Brøndum Frøkjær, Asbjørn Mohr Drewes

**Affiliations:** 1 Mech-Sense, Department of Gastroenterology and Hepatology, Aalborg University Hospital, Mølleparkvej 4, 9000 Aalborg, Denmark; d.bertoli@rn.dk (D.B.); e.mark@rn.dk (E.B.M.); dl@rn.dk (D.L.); t.okdahl@rn.dk (T.O.); s.nauser@rn.dk (S.N.); louise.daugberg@rn.dk (L.H.D.); christina.brock@rn.dk (C.B.); 2 Department of Clinical Medicine, Aalborg University, 9000 Aalborg, Denmark; jebf@rn.dk; 3 Steno Diabetes Center North Denmark, Aalborg University Hospital, Mølleparkvej 4, 9000 Aalborg, Denmark; 4 Department of Clinical Medicine, Faculty of Health and Medical Sciences, University of Copenhagen, 1353 Copenhagen, Denmark; birgitte.brock@regionh.dk (B.B.); filip.krag.knop.01@regionh.dk (F.K.K.); 5 Center for Clinical Metabolic Research, Gentofte Hospital, University of Copenhagen, 2900 Hellerup, Denmark; 6 Clinical Research, Steno Diabetes Center Copenhagen, 2730 Herlev, Denmark; 7 Department of Hepatology and Gastroenterology, Aarhus University Hospital, Mølleparkvej 4, 9000 Aalborg, Denmark; klaukrog@rm.dk; 8 Steno Diabetes Center Aarhus, 8200 Aarhus, Denmark; 9 Mech-Sense, Department of Radiology, Aalborg University Hospital, Mølleparkvej 4, 9000 Aalborg, Denmark

**Keywords:** magnetic resonance imaging, abdomen, gastrointestinal motility, gastrointestinal diseases, nausea

## Abstract

***Background:*** Diabetes-induced gastrointestinal (GI) symptoms are common but difficult to correctly diagnose and manage. We used multi-segmental magnetic resonance imaging (MRI) to evaluate structural and functional GI parameters in diabetic patients and to study the association with their symptomatic presentation. ***Methods:*** Eighty-six participants (46 with diabetes and GI symptoms, 40 healthy controls) underwent baseline and post-meal MRI scans at multiple timepoints. Questionnaires were collected at inclusion and following the scans. Data were collected from the stomach, small bowel, and colon. Associations between symptoms and collected data were explored. Utilizing machine learning, we determined which features differentiated the two groups the most. ***Key Results:*** The patient group reported more symptoms at inclusion and during MRI scans. They showed 34% higher stomach volume at baseline, 40% larger small bowel volume, 30% smaller colon volume, and less small bowel motility postprandially. They also showed positive associations between gastric volume and satiety scores, gastric emptying time and reflux scores, and small bowel motility and constipation scores. No differences in gastric emptying were observed. Small bowel volume and motility were used as inputs to a classification tool that separated patients and controls with 76% accuracy. ***Conclusions:*** In this work, we studied structural and functional differences between patients with diabetes and GI symptoms and healthy controls and observed differences in stomach, small bowel, and colon volumes, as well as an adynamic small bowel in patients with diabetes and GI symptoms. Associations between recorded parameters and perceived symptoms were also explored and discussed.

## 1. Introduction

Diabetes mellitus (DM) is a systemic disease affecting around 415 million people worldwide and is estimated to afflict over 600 million by 2040 [1]. The hyperglycemia and metabolic deterioration induced by DM are known to cause intracellular biochemical changes, oxidative stress, and local and systemic chronic inflammation [2]. These local alterations may lead to structural neuronal changes in the enteric nervous system that, together with alterations of glial cells, induce a remodeling of the gastrointestinal (GI) wall, resulting in reduced gastric compliance [3], reduced gastric contractility [4], impaired GI motility [5], and reduced secretions [6]. Taken together, these alterations are thought to be primary drivers of the underlying pathogenesis of GI symptoms [7]. The symptoms are most troublesome in patients with long-lasting, poorly controlled DM. They classically manifest as early satiety, nausea, vomiting, bloating, abdominal pain, flatulence, diarrhea, and constipation [8]. However, symptoms are often diffuse with atypical localizations, which may complicate diagnosis and treatment [9]. Hence, retrograde reflexes from lower GI segments may influence the secretory and myogenic activity in the upper gut and structural changes in the colon may result in nausea and vomiting. Diabetic enteropathy should therefore be considered a panenteric disorder presenting with segmental or panenteric dysmotility. As such, a multi-segmental examination is mandatory to provide comprehensive structural and functional measures to characterize GI symptom generation and maintenance in DM.

Due to its ability to non-invasively assess multiple parameters from multiple segments of the GI system, magnetic resonance imaging (MRI) has become increasingly popular [10]. Other examinations such as manometry, Wireless Motility Capsule, scintigraphy, and ultrasound imaging are routinely utilized to assess GI function in the clinical setting. However, these methods are limited by poor spatial resolution [11], long examination times [12], radiation exposure (scintigraphy), and readings that are poorly associated with symptoms [13]. Their limitations also restrict the usefulness of either of them as a single examination for panenteric assessment.

We hypothesized that MRI could, non-invasively, detect and quantify multiple structural (gastric, small bowel, and colonic volumes) and functional (gastric emptying, small bowel motility, and colonic water content) parameters throughout the GI system in subjects with diabetes. We, therefore, aimed (1) to develop and apply a multi-segmental MRI examination that could reliably assess segmental measures in patients with symptomatic diabetes and healthy volunteers; (2) to assess and describe relevant GI parameters before, during, and after a test meal; (3) to verify if any associations between the selected quantifiable parameters and experienced GI symptoms could be observed; and (4) to determine which measurable parameter separated the patients in our cohort from healthy patients.

## 2. Materials and Methods

### 2.1. Study Subject Selection

Data were obtained from 46 patients with diabetes and GI symptoms and 40 healthy volunteers without prior histories of diseases affecting the GI tract and not taking medication. Informed written consent was collected from all participants. Subjects were included in a larger clinical trial, described in the original published protocol from Okdahl et al. [14]. The study was registered at clinicaltrials.gov (NCT04143269) and was approved by the North Denmark Region Committee on Health Research Ethics (N-20190020). Inclusion criteria of the study were: (1) a diagnosis of type 1 or type 2 DM for a minimum of one year in stable treatment; (2) suspicion of diabetic autonomic neuropathy based on a minimum of one abnormal cardiovascular autonomic reflex test, or abnormal skin conductance, or a score above 16 in the COMPASS-31 questionnaire together with a combined weighted Gastroparesis Cardinal Symptom Index (GCSI) questionnaire and Gastrointestinal Symptom Rating Scale (GSRS) questionnaire score of minimum 2.3.

### 2.2. Study Design

A previously described panenteric MRI framework was utilized in this study [15]. In this paper, we will only briefly outline the methods.

### 2.3. Questionnaire Data

Information on upper and lower GI symptoms was collected at inclusion with the GCSI and GSRS questionnaires [16,17]. We included patients with diabetes based on a combined weighted score of GSRS and GCSI of a minimum of 2.3 to ensure a measurable symptomatic presentation. The cut-off for the weighted score was based on healthy cohorts as described in the work of Okdahl et al. [14]. Between the image acquisitions, participants were further asked to rate satiety, fullness, nausea, and pain using a numeric rating scale from 0 to 10 [18,19].

### 2.4. Image Acquisition

After 8 h of fasting, baseline images of the stomach, small bowel, and colon were acquired (see below). Then, after consuming a test meal (consisting of a 1:1 mixture of 200 mL of tap water and 200 mL of a 300 kcal protein shake, Nutridrik, Nutricia A/S, Lillerød, DK) [20], stomach and small bowel images were obtained again at 0 min, 15 min, 75 min, 90 min, and 105 min after meal intake. Colon images were captured again at 105 min. The participants maintained the supine position throughout the scan blocks. They were asked to briefly step out of the scanner close to the 0, 15, 75, and 105 min scan blocks to fill in a questionnaire.

### 2.5. MRI

MRI scans were performed using a 3T General Electric model SIGNA Premier (General Electric Medical Systems, Milwaukee, WI, USA). Stomach volume was assessed with an axial FIESTA (Fast Imaging Employing Steady-state Acquisition) sequence showing good contrast between liquid content and surrounding tissue. Stomach motility was assessed for 100 s with a dynamical FIESTA sequence with 1 slice going through the plane longitudinal to the gastric outlet where the distal gastric contraction waves were most visible.

Small bowel volume and motility were also assessed with a FIESTA sequence but in the coronal plane. Images for small bowel motility analysis were recorded for 20 s allowing for dynamical visualization of bowel contractions using 5 slices to comprehend the highest amount of small bowel volume possible.

The colonic volume was assessed with an SSFSE (Single Shot Fast Spin-Echo) sequence. The colonic water content (utilizing T1 relaxation times as a proxy) was evaluated through a coronal MOLLI (Modified Look-Locker Inversion Recovery) sequence. Details of each sequence can be seen in Supplementary File S1.

### 2.6. Quality Assessment

All images were anonymized and visually assessed for suitability. Unusable images were excluded from the analysis.

### 2.7. Image Processing

Following the previously proposed framework, gastric, small bowel, and colonic data were manually segmented as in previous studies [21]. After volume segmentation, gastric data were analyzed with a custom MATLAB framework (v.R2018b, MathWorks, Natick, MA, USA): the gastric total and compartmental volumes, normalized (to the total volume or area) compartmental volumes and surface areas, and gastric motility data were obtained [22]. The gastric half emptying time (T50) was calculated with the online tool ‘apps.menne-biomed.de/gastempt/’ [23].

Small bowel motility and volume data were analyzed using the online platform Entrolytics (Motilent, London, UK) [24,25]. The small bowel motility is expressed in arbitrary units (au.) with 0 as static. This surrogate measure reflects the metric used for this study, the standard deviation of the determinant of the pixel’s Jacobian averaged across a region of interest. This measure has been previously validated against clinical grading of motility [24,25].

Finally, colonic segmental volumes and water content (utilizing T1 relaxation times as a proxy) were obtained using a semiautomated MATLAB framework [26].

All segmented volumes (regardless of the program/platform utilized) were quantified as the sum of each segmented voxel multiplied by the voxel height, voxel width, and slice thickness.

### 2.8. Patient Classification Using Machine Learning

We analyzed our MRI and questionnaire data through supervised machine learning to select the best parameters that could divide the patient and the healthy group and thus observe if any form of clustering was present [27,28]. Firstly, the missing data in the dataset were imputed through multiple imputations by predictive mean matching [29]. Thirteen different datasets were generated, three were excluded at random, and one was chosen randomly from the remaining for the following analysis. Before analyses, the dataset was standardized. Elastic net linear regression was utilized to select the candidate features to feed the classifier algorithm. The feature selection, removing irrelevant data, and reducing dataset dimensionality is vital to increase learning accuracy and speed and improve the comprehensibility of results. The elastic net regression, as the name implies, is a regularized regression model that utilizes a combination of both ridge and lasso penalties to best fit the model. From the complete dataset, 70% of the data (randomly selected) was utilized for training the elastic net regression model, while the remaining 30% was used to assess its precision. The selected features, the best tuning hyperparameter obtained via repeated cross-validation, and the regression model performances were reported. To observe if any form of clustering was present in our data, we utilized a classifier algorithm, the linear discriminant analysis, which, not differently from logistic or probit regressions, attempts to express one categorical dependent variable (the presence of diabetes, in our case) as a linear combination of other features. Linear discriminant analysis explicitly attempts to model the differences between classes of data, projecting features into a lower dimension space by introducing a new latent variable that maximizes the distance between the means of the two classes and minimizes the variation within each class. Linear discriminant analysis was performed on a dataset containing all the identified features. From this dataset, 70% of data (randomly selected) was utilized for the training of the classifier algorithm, while the remaining 30% was used to assess the model’s performance. A confusion matrix with the predicted and observed observations was generated, and marginal frequencies were tested with McNemar’s test. The classifier model performances were reported. All the analyses were performed after data seeding to ensure the reproducibility of the results.

### 2.9. Statistical Analysis

Data were analyzed in R (R Core Team, 2021, R Foundation for Statistical Computing, Vienna, Austria) [30]. After identifying extreme outliers (defined as values above the third quartile + 3 interquartile ranges or below the first quartile–3 interquartile ranges), data were inspected for normality through Q–Q plots and Shapiro–Wilk tests. Extreme outliers were managed by reviewing the underlying data and removing them during post-test analyses if due to technical errors, which we believe to be, in our study, the best tradeoff to preserve the highest amount of data while not neglecting the role of outliers in our sample and in a clinical environment. Non-normally distributed data were normalized through Box-Cox transformation. Differences between the two groups were assessed with one-way repeated measure ANOVA, tested for sphericity with Mauchly’s test, and corrected with Greenhouse–Geisser and Huynh–Feldt corrections [31]. In case of significance, post hoc analyses were performed with pairwise *t*-tests adjusted with sequential Bonferroni tests (Bonferroni–Holm) [32,33]. Associations in colonic data were assessed through paired-sample *t*-tests, as only 2 data points were present.

Correlations between questionnaire data and other representative parameters were reported through Spearman’s rank correlation coefficients, as questionnaire data were rank-ordered, and the eventual relationship with parametric data was expected to be monotonic. As GCSI and GSRS questionnaires were filled in by the subject while fasting, only parameters at baseline were tested for associations. It was chosen to only test the most representative parameters for each segment, describing either its structure or its function. It was therefore chosen to test stomach volume and T50, small bowel volume and motility, colonic volume, and T1 values.

Data are reported as mean (SD). *p* values < 0.05 were considered to be statistically significant. The R packages used are reported in Supplementary File S1.

## 3. Results

### 3.1. Demography

All recruited subjects complied with the study protocol, and a complete dataset was obtained from most participants (240 data points corresponding to 4.5% of data were either missing or excluded after quality control). For the complete demographic data, see Table 1.

### 3.2. Questionnaires

#### 3.2.1. Symptoms Assessed at Baseline

Compared to healthy participants, participants with diabetes had higher scores of nausea, satiety, and bloating (all *p* < 0.001) as measured with the GCSI questionnaire. Likewise, the diabetes group also had higher scores of reflux, abdominal pain, indigestion, diarrhea, and constipation (all *p* < 0.001) measured with the GSRS questionnaire. For details, see Table 1.

#### 3.2.2. Symptom Scores in Response to a Test Meal

**Figure 1:**
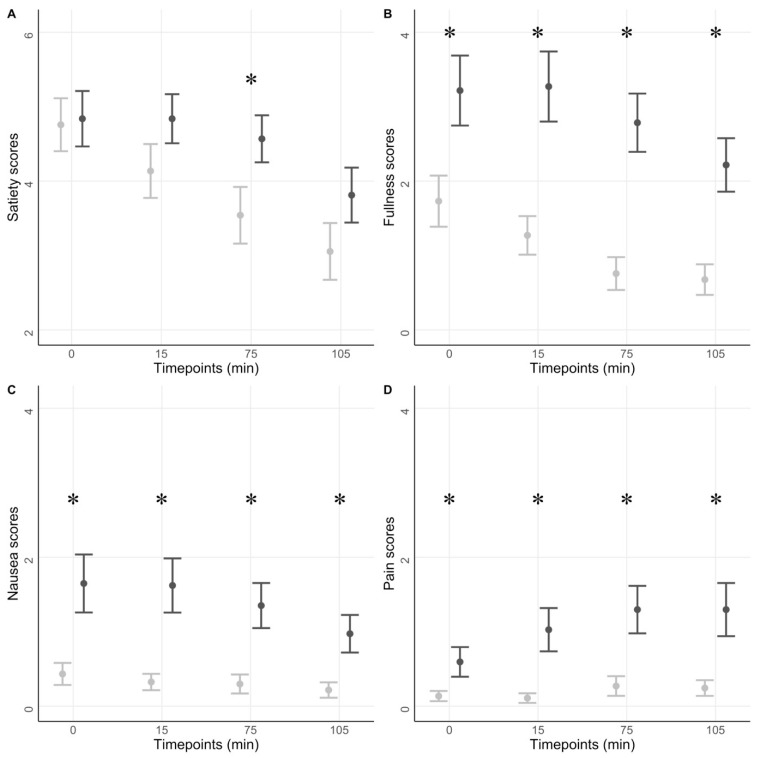
GI symptoms scores. The figure shows questionnaire data for the diabetic group (dark grey) and the healthy volunteers (light grey). A liquid meal was ingested at 0 min. The four scores, satiation (**A**), fullness (**B**), nausea (**C**), and abdominal pain (**D**), are reported. Means and SE are shown. * *p* < 0.05.

A group difference in the numerical rating scale values was observed for satiety, fullness, nausea, and pain scores (all *p* < 0.001). Post hoc analyses showed a significant difference in satiety scores at 75 min between the two groups (*p* = 0.04). Here, the diabetes group showed a mean score of 4.3 ± 1.8, whereas the healthy group scored 3.6 ± 2.5. Furthermore, the diabetes group showed significantly higher values in fullness, nausea, and pain scores than the healthy group at every timepoint (all *p* < 0.05). See Figure 1.

The complete dataset can be found in Supplementary File S2, Table S5.

### 3.3. Gastric Volumes Distribution and Compartmental Analysis

**Figure 2:**
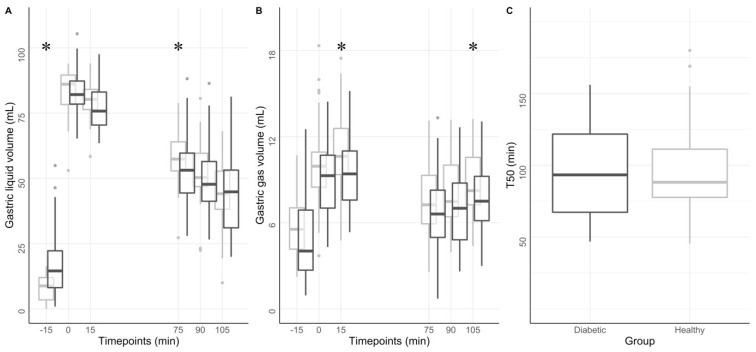
Gastric volumes and T50. The figure shows gastric liquid (**A**) and gas volumes (**B**) at different timepoints for the diabetic group (dark grey) and the healthy group (light grey). A liquid meal was ingested at timepoint 0 min. Gastric half—emptying time (T50) distributions (**C**). * *p* < 0.05.

A difference in total gastric volumes between groups was only observed at baseline, where the volume in the diabetic group was higher than in the healthy group, 203 ± 89 mL vs. 152 ± 41 mL, *p* < 0.005. This difference was associated with a significantly higher liquid gastric volume in the diabetic group, 43 ± 58 mL vs. 21 ± 20 mL, *p* < 0.001. The volumetric difference was predominantly seen in the corpus, with 108 ± 52 mL in the diabetic group and 72 ± 28 mL in the healthy group (*p* = 0.001), and in the antrum, with 71 ± 35 mL in the diabetic group and 57 ± 18 mL in the healthy group (*p* = 0.04). These differences were also observed on the surface of the same compartments: the surface area of corpus showed higher values in the diabetic group (156 ± 80 cm^2^) than in the healthy group (101 ± 25 cm^2^), *p* < 0.001. Antrum surface area showed a similar distribution, though not as substantial: 120 ± 40 cm^2^ for the diabetic group and 100 ± 24 cm^2^ in the healthy volunteers, *p* > 0.05. A difference in gastric wall deformation was observed at 0, 15, 75, 90, and 105 min in the antrum (all *p* < 0.05), showing less deformation in the patient group as compared with the healthy group. Deformations in the fundus and corpus showed no significant differences between groups at every timepoint (Figure 2A,B). The complete dataset can be found in Supplementary File S2, Table S1, Table S2(a–c).

### 3.4. Gastric Motility and Emptying

No difference in gastric contraction frequency was observed between the two groups, with 3.3 ± 0.5 contractions/min in the diabetic group and 3.2 ± 0.6 in the healthy group (*p* > 0.5). Likewise, no significant difference in gastric emptying was observed (*p* > 0.05). The gastric emptying half-time showed a value of 94 ± 31 min in the diabetic group and 95 ± 30 min in the healthy group, Figure 2C.

### 3.5. Small Bowel Volume and Motility

**Figure 3:**
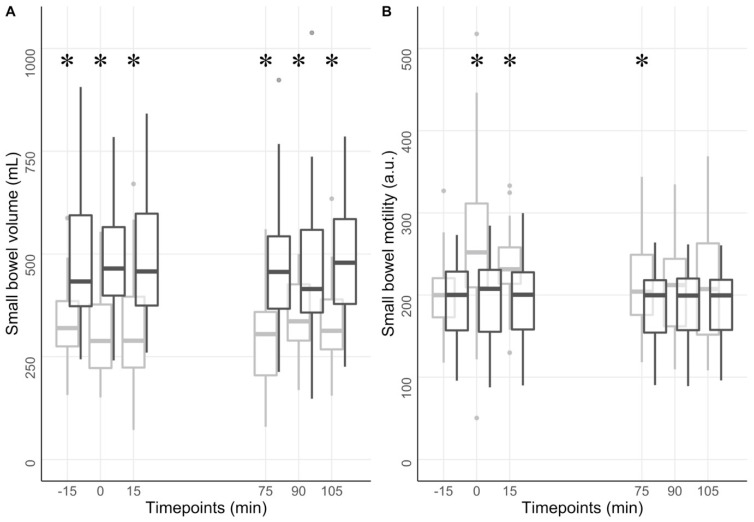
Small bowel volumes and motility. The figure shows small bowel volume (**A**) and motility (**B**) at different timepoints for the diabetic group (dark grey) and healthy group (light grey). A liquid meal was ingested at timepoint 0 min. * *p* < 0.05.

We showed larger small bowel volumes at every timepoint (all *p* < 0.001) in patients with diabetes compared to controls. Small bowel volume ranged from 490 ± 142 mL at 15 min to 463 ± 154 mL at 90 min in the diabetic group and from 350 ± 171 mL to 334 ± 101 mL at the same timepoints in the healthy volunteers. We observed lower motility scores in the diabetic group at 0, 15, and 75 min compared to healthy volunteers (all *p* < 0.05). The motility score ranged from 201 ± 39 au at baseline to 199 ± 48 au at 0 min in the diabetic group. At the same timepoints, values ranged from 204 ± 49 au to 282 ± 99 au in the healthy volunteer group (Figure 3).

The complete dataset can be found in Supplementary File S2, Table S3.

### 3.6. Colonic Volume and Motility

**Figure 4:**
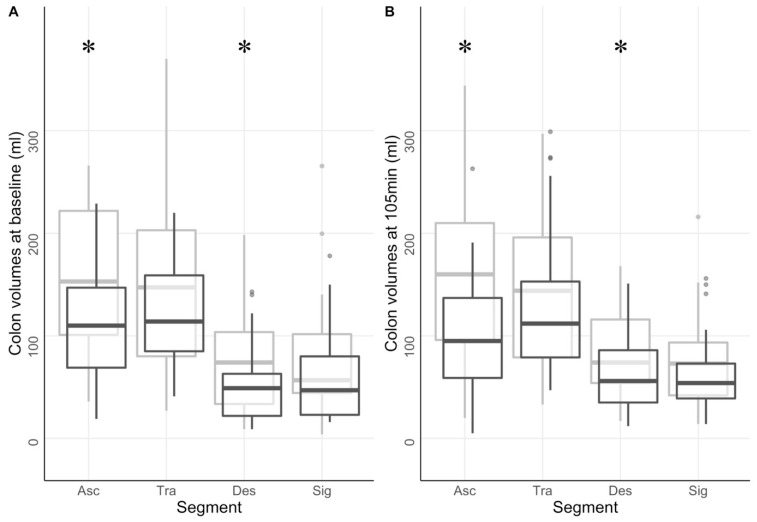
Colonic volumes. The figure shows colon segmental volumes at baseline (**A**) and 105 min (**B**) for the diabetic group (dark grey) and the healthy group (light grey). * *p* < 0.05.

Analysis of colonic segmental volumes showed lower total colonic volume in the diabetes group compared to healthy volunteers (*p* = 0.02). The diabetes group ranged from 375 ± 209 mL at baseline to 398 ± 214 mL at 105 min. Volumes in the healthy group ranged from 521 ± 327 mL to 529 ± 260 mL at the same timepoints. Differences in volume between groups were observed at both baseline and 105 min in ascending colon (*p* < 0.006 and *p* < 0.001) and in descending colon (*p* < 0.02 and *p* < 0.05), Figure 4.

The complete dataset can be found in Supplementary File S2, Table S4(a,b).

### 3.7. Colonic Water Content

No differences in T1-relaxation times were observed between the two groups (all *p* > 0.05). T1-times ranged from 715 ± 123 ms at baseline to 680 ± 154 ms at 105 min in the healthy group. The patient group ranged from 648 ± 183 ms to 683 ± 182 ms at the same timepoints. The complete dataset can be found in Supplementary File S2, Table S4(a).

### 3.8. Association between Symptoms Scores and Multi-Segmental MRI Measures

In the diabetic group, a significant association was observed between the total stomach volume and satiety score (from the GCSI questionnaire) (*p* < 0.005, Rho = 0.414), while reflux scores (also from the GCSI questionnaire) associated with gastric emptying half-times (*p* = 0.01, Rho = −0.432). Furthermore, constipation scores (from the GSRS questionnaire) were significantly associated with small bowel motility in the diabetic group (*p* < 0.005, Rho = 0.466). These associations were not found in the healthy group. No associations between questionnaires submitted during the examination and observed parameters were observed (all *p* > 0.05).

### 3.9. Elastic Net Regression and Linear Discriminant Analysis

**Figure 5:**
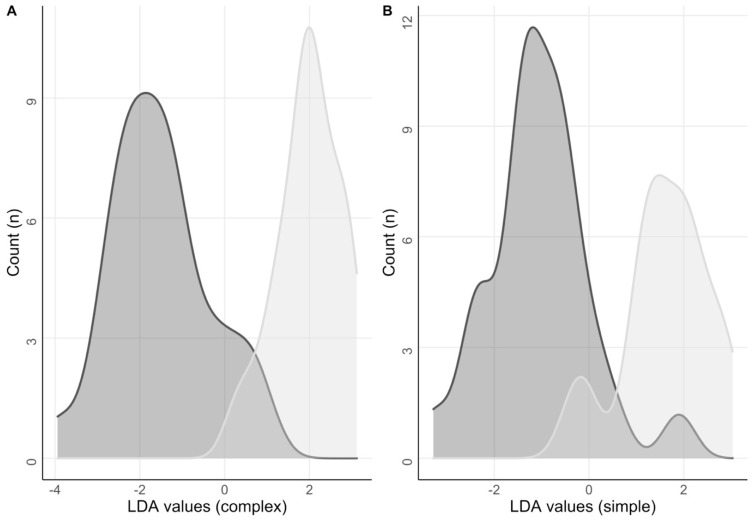
Linear Discriminant Analysis (LDA). The figure shows different LDA values and their relative frequencies for the diabetic group (dark grey) and the healthy group (light grey) for both the complex (**A**) and simplified model (**B**).

The missing data points (4.5% of the total data) were imputed before performing the following analyses. After training the elastic net regression model on the whole dataset, the best tuning parameters observed were: α = 0.11 and λ = 0.5, which moved the model closer to a ridge than a lasso. Utilizing these parameters, the model identified 9 variables as potential features: Gastric gas content at 105 min, small bowel volume at 0, 75, and 105 min, small bowel motility at 0 and 15 min, and fullness and nausea scores at 75 and 105 min. The observed performance was: root-mean-square error 1.04 ± 0.19, R^2^ 0.83 ± 0.17, mean absolute error 1.00 ± 0.18. Using these variables as features for a cluster analysis, the classifier algorithm correctly classified 83% of the test data into their original class (diabetics or controls). The performance observed was: accuracy 0.857, 95% CI 0.571–0.982, *p*-value 0.02, sensitivity 0.875, specificity 0.833, positive predictive value 0.875, and negative predictive value 0.833. McNemar’s test showed a *p* = 1 (Figure 5A).

As small bowel data bore the highest weight in the model (see Supplementary File S2, Table S6), we used the same algorithm, selecting only small bowel-related features, sacrificing performance scores for interpretability. Using only small bowel volume at 0 and 105 min and motility at 0 and 15 min, the classifier algorithm correctly classified 76% of the test data into their original classes. The performance observed was: accuracy 0.764, 95% CI 0.501–0.931, *p*-value 0.04, sensitivity 0.625, specificity 0.889, positive predictive value 0.833, and negative predictive value 0.727. McNemar’s test showed a *p* = 0.6 (Figure 5B).

## 4. Discussion

Using a novel multi-segmental MRI framework, we observed several differences between patients and a healthy volunteer population. While previous studies have reported MRI examinations of several segments of the gut, we added more structural and functional details with the multi-segmental gut examination resulting in a detailed panenteric characterization of the gut [10,34,35]. We observed significant differences in static parameters as diabetic patients showed lower stomach volumes at baseline, characterized by lower volumes in the fundus and antrum. The diabetic group also showed higher small bowel volumes and lower colon ascendent and descendent volumes during the whole examination. Differences in functional parameters were also observed: the diabetic group showed no differences in gastric emptying and colonic water content with the healthy group. It showed, however, a lack of small bowel motility response to the meal, with lower scores up to 75 min postprandially. Furthermore, associations between total stomach volume and satiety, reflux scores and gastric emptying half-times, and constipation scores and small bowel motility were observed in the diabetic group. With machine learning, we observed a clustering of diabetic patients predominantly due to small bowel volumes and motility scores.

### 4.1. Overview of Findings and Comparison with Recent Literature

Our data on healthy subjects remained within the expected normal range [10,36,37,38,39]. Furthermore, the comorbidities of the DM group at inclusion observed in this study also fall within the range expected from the literature in patients 5+ years after diagnosis [40], which would indicate that GI symptoms due to diabetes could be expected.

#### 4.1.1. Stomach

In accordance with recent literature, we observed that patients with diabetes showed higher fasting gastric volumes compared to healthy volunteers, while no differences could be observed after meal intake [41], suggesting that gastric compliance was unhindered in our cohort [3]. Furthermore, we observed no group differences in gastric emptying time and motility. However, increased small bowel volume and decreased motility patterns were observed in diabetes patients. Our findings are supported by numerous studies questioning the classical focus on gastric emptying and the presence of enteric dysmotility in DM [42,43,44,45]. Indeed, a recent investigation based on breath tests and small bowel manometry showed that only 28% suffered from delayed gastric emptying, 80% suffered from small bowel abnormalities, and enteric dysmotility was present in 96% of those with delayed gastric emptying [46]. The findings led the authors to conclude that “gastroparesis” is likely associated with small bowel motility rather than being the primary disease. Furthermore, even if the diabetic cohort showed consistently higher symptom scores across every questionnaire item in our study, the post-meal gastric volume and motility did not differ significantly from the controls. This highlights that symptoms are not related to the dynamics of the emptying phase, consistent with the poor association between delayed gastric emptying and symptomatology, as underlined in a recent United European Gastroenterology and European Society for Neurogastroenterology and Motility consensus paper on gastroparesis [47].

#### 4.1.2. Small Bowel

We observed that the small bowel in the diabetic group was adynamic in response to the ingested meal, indicating impaired reflexes. The findings are supported by other studies, which have shown diabetes-induced duodenal hypomotility as part of the pylorospasm complex [48], inadequate response to a meal up to one hour postprandial [49,50], and evident as prolonged small bowel or orocecal transit time [51]. Small bowel volume was increased at all timepoints, and the finding was so specific that, when used in the classification model to discriminate between patients with diabetes and healthy patients, small intestine data alone performed with a 76% classification accuracy, which improved to 83% classification accuracy in the more complex model also comprehending numeric rated scale and gastric volume data. In recent literature, there have been diverging findings on the effect of diabetes on small bowel motility and transit time [52,53,54]. Our observations point toward a transit time prolongation, consistent with most literature on the topic. Furthermore, previous reports support our observations on small bowel volume, which we found higher in the diabetic group than in the healthy control group [55]. At last, the diabetic group showed a correlation between small bowel motility and constipation scores, also in agreement with the recent literature on the subject [56].

#### 4.1.3. Colon

In our MRI-based model, colonic volumes were smaller in participants with diabetes compared to healthy participants, which showed volumes comparable with the recent literature [26], and with no differences in water content. The finding is surprising as colonic transit times, e.g., assessed with the wireless motility system, are prolonged in patients with type 1 diabetes. Prolonged transit time may lead to the accumulation of feces in the colon and have previously been linked to lower T1-relaxation times [26]. However, this cohort was comprised of people with diabetes and verified polyneuropathy, whereas our patients were included based on GI symptoms. Thus, the two results may not contradict each other, as recent literature shows a poor correlation between constipation symptoms and colonic volumes and transit times [57]. Nevertheless, studies in recent literature seem to support our findings, with healthy volunteers showing smaller, though not statistically significant, colonic volumes than diabetic patients [58]. Recent studies have challenged the correlation between colon volume and constipation symptoms, as patients with irritable bowel syndrome constipation type showed lower colon volumes than patients with functional constipation. These observations highlight the subjective experience of GI symptoms, probably influenced by different confounding factors such as other diseases, concomitant drug use, or psychological factors. In our study, no associations between symptoms and colonic volumes or colonic water content were observed.

From a mechanical standpoint, it would appear that, in the diabetic group, a redistribution of volumes toward the small bowel occurred, possibly due to diminished small bowel motility and therefore diminished liquid outflow in the direction of the colon. However, it cannot be excluded that changes in the absorption and secretion of fluids due to enteric neuropathy may influence our findings. These differences were underlined and magnified by the linear discriminant analysis, which observed a decided clustering of the two populations.

### 4.2. Limitations and Future Perspectives

This study has limitations. As in many MRI studies, long post-processing times are required to analyze multiple parameters in the GI system. This requirement limits the applicability of this method in a clinical setting, although future technical developments will likely partially automatize the analysis. Furthermore, we evaluated small bowel motility from a merely geometric point of view, measuring the motility as the geometrical changes in the region of interest, including contraction and expansion of the bowel wall. This method cannot precisely describe the directionality of contractions: non-propulsive waves are estimated to be as high as propulsive ones of equal amplitude. While the ability to measure the motility of the small bowel, as demonstrated in this work, is vital for understanding GI functions as a whole, we still lack essential insight into this process. Our measurements of small bowel volume are also based on the segmentation of five slices of one centimeter each, which tends to underestimate volumes at higher BMIs, where a portion of the small bowel falls outside our visible region. This problem is even more relevant in diabetic patients, who are prone to higher BMIs than healthy controls, as observed in our demographic data. Participants with diabetes were heterogeneous, consisting of people with different types of diabetes and different symptoms of the autonomic nervous system. However, all participants experienced GI symptoms ranging from mild to moderate to severe, and a group analysis based on this could have revealed other gut segmental findings. At last, we did not include diabetic patients without GI symptoms nor a group without diabetes and with GI symptoms. Due to this design choice, we cannot generalize our results to the entire diabetic population. However, we have previously observed that gut sensations in diabetics are often “silent” due to neuropathy-induced general hypoalgesia, coupled with central hyperexcitability [3]. Therefore, subjective symptoms are unreliable in this context. Furthermore, a group of diabetics without gastrointestinal symptoms may be difficult to identify, as the prevalence of gastrointestinal symptoms can surpass 70% in many outpatient samples [59]. Despite these limitations, further studies are needed to assess if the observed results are common in diabetes or if they are exclusive of diabetic gastroenteropathies.

### 4.3. New Directions

In this study, our objective was to comprehensively observe and report the functional and mechanical presentation of diabetes with gastrointestinal symptoms. However, it is essential to acknowledge that various other factors, beyond the scope of this work, contribute to the genesis, development, and clinical presentation of diabetes. Recent studies have explored further areas, e.g., the role of autoimmunity in transmission disturbances of the migrating myoelectric complex [60]. Other research has examined the role of gut microbiota and the products of their metabolism on the electric, neuronally mediated, communications in the distal colon [61]. The roles of endothelial disfunction and micro-RNAs have also shown an influence on the pathogenesis of motor disturbances [62,63]. However, future studies are still necessary to identify potential therapeutic targets.

## 5. Conclusions

In this work, diabetes, in a cohort included based on GI symptoms, was associated with objective, quantifiable changes in GI functions and postprandial symptoms compared to healthy controls. We have examined and assessed several volumetric and motility-related features of different GI segments, outlining their association with the symptomatic presentations of diabetic patients. Through machine learning, we observed how the small bowel appeared to play a significant role in the clustering of diabetic patients. Furthermore, we reported how constipation, a symptom classically correlated to the colon and its functions, was strongly associated with the small bowel instead. This observation highlights the importance of a panenteric approach in evaluating complex GI symptomatic presentations, as those generally observed in diabetic patients.

However, further studies are necessary to understand GI symptoms and depict in detail the differences between the two groups and the underlying disease mechanisms. This would benefit patients, allowing us to monitor treatment effects and disease evolution.

## Figures and Tables

**Table 1:** ***Demography.*** The table shows the demographic baseline data of the diabetic and healthy control groups. Gastroparesis Cardinal Symptom Index (GCSI) and Gastrointestinal Symptom Rating Scale (GSRS) mean (SD) subscores are shown. * *p* < 0.001.

	Patients with DM and GI Symptoms (n = 46)	Healthy (n = 40)
Age (years)	62 ± 12	57 ± 10
Gender (M/F)	28:18	27:13
BMI * (kg/m^2^)	30 ± 6	27 ± 5
Smokers (%)	9.5 (n = 4)	8.1 (n = 3)
Mean systolic pressure (mmHg)	134 ± 15	141 ± 21
Mean diastolic pressure *	78 ± 9	85 ± 11
Diabetes (type1/type2)	9:36	-
Diabetes duration (years)	20 ± 14	-
Charlson Comorbidity index	1.8 ± 1.0	-
*Questionnaires-GCSI*		
Nausea score *	0.7 ± 0.8	0 ± 0
Satiety score *	1.5 ± 0.9	0.3 ± 0.5
Bloating score *	1.9 ± 1.5	0.3 ± 0.6
Total GCSI score *	1.4 ± 0.9	0.3 ± 0.4
*Questionnaires-GSRS*		
Reflux score *	2.1 ± 1.0	1.3 ± 0.6
Abdominal pain score *	2.5 ± 1.0	1.1 ± 0.2
Indigestion score *	3.2 ± 1.4	1.3 ± 0.4
Diarrhea score *	3.0 ± 1.6	1.1 ± 0.3
Constipation score *	3.3 ± 1.4	1.1 ± 0.2
Total GSRS score *	2.8 ± 0.8	1.2 ± 0.2

## Data Availability

The data presented in this study are available on request from the corresponding author.

## Supplementary Materials

The following supporting information can be downloaded at: https://www.mdpi.com/article/10.3390/jcm12185968/s1, Table S1: Gastric volume data; Table S2(a–c): Gastric compartments volume, surface area, inverse curvature; Table S3: Small bowel volume and motility data; Table S4(a): Colon volume and water content; Table S4(b): Colon segments volume; Table S5: Questionnaires data; Table S6: LDA coefficients.
