# Supplementary material for: MRI-Based Quantification of Pan-Alimentary Function and Motility in Subjects with Diabetes and Gastrointestinal Symptoms
Source: J Clin Med. 2023 Sep 14;12(18):5968. doi: 10.3390/jcm12185968 (PMC10532375; doi:10.3390/jcm12185968)
Supplement: Supplementary file 1 [file jcm-12-05968-s001.zip › Supplementary File S1.pdf]

R packages utilized

The following R packages were utilized in this work:

-readxl
-ggplot2
-dplyr
-tidyvers
-ggpubr
-rstati
-onewaytests
-multcomp
-readr
-MASS
-lattice
-caret

MRIs

MRI scans were performed using a 3T General Electric model SIGNA Premier (General Electric Medical Systems, Milwaukee, WI, USA). Stomach volume and motility were assessed with an axial FIESTA (Fast Imaging Employing Steady-state Acquisition) sequence, echo time (TE): 1.2 ms, repetition time (TR): 2.8 ms, flip angle: 45°, slice thickness: 5.0 mm, slice spacing: 0 mm, number of slices: 30-40, acquisition matrix: 160/0/0/200, reconstruction diameter: 420 pixels, percent phase field of view: 100, columns: 512, columns: 512. Volume images were recorded in breath-hold. Motility scans were performed with an initial 20-second breath-hold followed by 100 seconds of free breathing. These scans were acquired in the plane where the gastric contraction waves were more visible.

Small bowel volume and motility utilized the same sequence as the stomach but in the coronal plane. Images were recorded using 5 slices to comprehend the highest amount of small bowel volume possible; each slice had a scanning time of 1 minute, the first 20 seconds in breath-hold.

The colonic volume was assessed with an SSFSE (Single Shot Fast Spin-Echo) sequence, TE: 90 ms, TR: automatically calculated, set to ”minimum”, flip angle: 90°, slice thickness: 4.0 mm, slice spacing: 0 mm, number of slices: 35-40, acquisition matrix: 0/320/256/0, reconstruction diameter: 500 pixels, percent phase field of view: 100, columns: 512, rows: 512. The colonic T1 relaxation time (T1-mapping) was assessed through a MOLLI (Modified Look-Locker Inversion Recovery) sequence, TE: 1.2 ms, TR: 2.9 ms, flip angle: 35°, slice thickness: 6.0 mm, slice spacing: 0 mm, number of slices: 3, acquisition matrix: 0/160/148/0, reconstruction diameter: 360 pixels, percent phase field of view: 75, columns: 256, rows: 256.
