# Supplementary material for: MRI-Based Quantification of Pan-Alimentary Function and Motility in Subjects with Diabetes and Gastrointestinal Symptoms
Source: J Clin Med. 2023 Sep 14;12(18):5968. doi: 10.3390/jcm12185968 (PMC10532375; doi:10.3390/jcm12185968)
Supplement: Supplementary file 1 [file jcm-12-05968-s001.zip › Supplementary File S2.pdf]

Table S1

Time	Gastric total volume (ml)		Gastric liquid volume (ml)		Gastric gas volume (ml)	
	Diabetes	*Healthy*	*Diabetes*	*Healthy*	*Diabetes*	*Healthy*
Baseline	203 (89)	152 (41)	43 (58)	21 (20)	24 (28)	23 (14)
0 min	569 (108)	559 (91)	416 (71)	420 (66)	55 (34)	73 (49)
15 min	543 (104)	548 (82)	385 (68)	392 (73)	60 (33)	81 (46)
75 min	359 (107)	351 (73)	222 (84)	245 (60)	35 (29)	44 (30)
90 min	341 (103)	326 (79)	203 (84)	214 (58)	39 (32)	48 (31)
105 min	323 (102)	291 (71)	167 (85)	168 (69)	42 (32)	51 (26)

Table S1. ***Gastric volume data***. The table shows total gastric volume (including the gastric wall volume), liquid volume and gas volumes. Data are reported as mean (SD).

Table S2.a

Time	Fundus Volume (ml)		Corpus Volume (ml)		Antrum volume (ml)	
	Diabetes	*Healthy*	*Diabetes*	*Healthy*	*Diabetes*	*Healthy*
Baseline	24 (15)	23 (16)	108 (52)	72 (28)	71 (35)	57 (18)
0 min	141 (57)	161 (78)	264 (82)	237 (84)	163 (60)	162 (51)
15 min	131 (56)	139 (55)	252 (71)	251 (81)	160 (56)	158 (46)
75 min	53 (30)	70 (33)	186 (68)	163 (50)	120 (51)	118 (33)
90 min	49 (33)	62 (35)	173 (66)	152 (56)	119 (47)	112 (31)
105 min	44 (30)	48 (33)	163 (62)	132 (48)	116 (54)	111 (28)

Table S2.b

Time	Fundus Surface Area (cm^2)		Corpus Surface Area (cm^2)		Antrum Surface Area (cm^2)	
	Diabetes	*Healthy*	*Diabetes*	*Healthy*	*Diabetes*	*Healthy*
Baseline	39 (20)	37 (15)	156 (80)	101 (25)	120 (40)	100 (24)
0 min	120 (38)	129 (44)	218 (71)	179 (46)	194 (44)	191 (39)
15 min	112 (37)	117 (35)	210 (75)	187 (48)	193 (39)	188 (36)
75 min	64 (31)	71 (25)	193 (72)	149 (34)	159 (43)	161 (30)
90 min	56 (27)	65 (24)	186 (71)	145 (38)	161 (39)	159 (37)
105 min	52 (25)	55 (24)	180 (70)	136 (38)	158 (48)	158 (31)

Table S2.c

Time	Fundus Inverse Curvature (mm^{-1})		Corpus Inverse Curvature (mm^{-1})		Antrum Inverse Curvature (mm^{-1})	
	Diabetes	*Healthy*	*Diabetes*	*Healthy*	*Diabetes*	*Healthy*
Baseline	8.9 (1.3)	8.7 (1.5)	8.5 (1.4)	8.0 (1.6)	7.0 (1.0)	7.0 (1.0)
0 min	11.5 (1.6)	10.8 (2.0)	9.8 (1.6)	9.5 (1.6)	7.8 (1.2)	8.4 (1.0)
15 min	11.9 (2.1)	11.0 (1.5)	10.0 (1.6)	9.7 (1.5)	7.7 (1.1)	8.4 (1.0)
75 min	9.9 (1.1)	10.3 (1.1)	9.4 (1.4)	9.3 (1.2)	7.5 (0.9)	8.1 (1.0)
90 min	9.9 (1.7)	10.2 (1.4)	9.2 (1.4)	9.0 (1.4)	7.3 (1.0)	7.9 (0.8)
105 min	10.2 (1.4)	9.6 (1.5)	9.3 (1.4)	9.0 (1.4)	7.4 (1.1)	7.8 (1.2)

Table S2.a, A2.b, A2.c. ***Gastric compartments volume, surface area, inverse curvature***. The tables show volumes, surface areas, and inverse curvatures of fundus, corpus, and antrum.

Table S3

Time	Volume (ml)		Motility (a.u.)	
	Diabetes	*Healthy*	*Diabetes*	*Healthy*
Baseline	474 (161)	348 (84)	201 (39)	204 (49)
0 min	482 (121)	304 (103)	199 (48)	282 (99)
15 min	490 (143)	350 (171)	195 (45)	228 (45)
75 min	468 (157)	299 (108)	190 (45)	215 (50)
90 min	463 (154)	334 (101)	191 (46)	206 (60)
105 min	475 (135)	323 (79)	191 (47)	207 (72)

Table S3. ***Small bowel volume and motility data***. The table shows total small bowel volume and motility.

Table S4.a

Time	Total colonic volume (ml)		T1-relaxation time, Ascending Colon (ms)		T1-relaxation time, Descending Colon (ms)	
	Diabetes	*Healthy*	*Diabetes*	*Healthy*	*Diabetes*	*Healthy*
Baseline	375 (209)	521 (328)	648 (189)	715 (123)	609 (200)	606 (179)
105 min	398 (214)	529 (260)	683 (182)	680 (154)	584 (234)	599 (142)

Table S4.a. ***Colon volume and water content***. The table shows total colonic volume and water content (expressed as T1 relaxation times).

Table S4.b

Time	Ascending Colon Volume (ml)		Transverse Colon Volume (ml)		Descending Colon Volume (ml)		Sigmoid Colon Volume (ml)	
	Diabetes	*Healthy*	*Diabetes*	*Healthy*	*Diabetes*	*Healthy*	*Diabetes*	*Healthy*
Baseline	112 (55)	156 (70)	121 (50)	157 (90)	52 (36)	78 (48)	57 (41)	76 (54)
105 min	102 (56)	161 (81)	129 (69)	149 (75)	62 (34)	84 (44)	62 (35)	77 (46)

Table S4.b. ***Colon segments volume***. The table shows colonic volume of the four colon segments.

Table S5

Time	Satiety		Nausea		Fullness		Pain	
	Diabetes	*Healthy*	*Diabetes*	*Healthy*	*Diabetes*	*Healthy*	*Diabetes*	*Healthy*
0 min	4.8 (2.1)	4.9 (2.3)	3.4 (2.9)	1.8 (2.2)	1.6 (2.3)	0.4 (0.8)	0.8 (1.5)	0.1 (0.5)
15 min	4.7 (2.0)	4.3 (2.2)	3.3 (2.9)	1.2 (1.6)	1.7 (2.2)	0.3 (0.6)	1.2 (1.8)	0.1 (0.3)
75 min	4.3 (1.8)	3.6 (2.5)	2.9 (2.4)	0.6 (1.3)	1.4 (1.9)	0.1 (0.4)	1.4 (2.0)	0.1 (0.5)
105 min	3.6 (2.2)	3.2 (2.5)	2.3 (2.2)	0.6 (1.2)	1.0 (1.6)	0.1 (0.5)	1.4 (2.2)	0.2 (0.5)

Table S5. ***Questionnaires data***. The table shows the questionnaires' scores on Satiety, Fullness, Nausea, and Abdominal Pain as rated on a 0-10 visual analogue scale, where 0 is no symptoms.

Table S6

Variable name	Coefficients absolute values
Small bowel volume 0 min	0.72
Small bowel volume 105 min	0.41
Small bowel motility 0 min	0.25
Small bowel motility 15 min	0.20
Stomach gas volume at 105 min	0.04
Fullness score NRS 75 min	0.23
Fullness score NRS 105 min	0.30
Nausea score NRS 75 min	0.07
Nausea score NRS 105 min	0.01

Table S6. ***LDA coefficients***. The table shows the coefficients of the variables utilized as features by the classifier. Absolute values are reported. NRS (numeric rated scale).
